# Capture of mobile genetic elements following intercellular conjugation promotes the production of ST11-KL64 CR-hvKP

**DOI:** 10.1128/spectrum.01347-24

**Published:** 2025-02-03

**Authors:** Shanshan Huang, Dan Dan Wei, Hanxu Hong, Si Chen, Lin-Ping Fan, Qi-Sen Huang, Fang-Ling Du, Tian-Xin Xiang, Ping Li, Wei Zhang, La-Gen Wan, Yang Liu

**Affiliations:** 1 Department of Clinical Microbiology, The First Affiliated Hospital, Jiangxi Medical College, Nanchang University, Nanchang, China; 2 National Regional Center for Respiratory Medicine, China-Japan Friendship Hospital Jiangxi Hospital, Nanchang, China; 3 Department of Infectious Disease, The First Affiliated Hospital, Jiangxi Medical College, Nanchang University, Nanchang, China; 4 Department of Respiratory and Critical Care Medicine, The First Affiliated Hospital, Jiangxi Medical College, Nanchang University, Nanchang, China; 5 Department of Clinical Laboratory, Medical Center of Burn Plastic and Wound Repair, The First Affiliated Hospital, Jiangxi Medical College, Nanchang University, Nanchang, China; 6 Jiangxi Medicine Academy of Nutrition and Health Management, Nanchang, China; City of Hope Department of Pathology, Duarte, California, USA

**Keywords:** *Klebsiella pneumoniae*, CR-hvKP, mobile genetic elements, conjugation, fitness costs

## Abstract

Sequence type (ST)11 carbapenem-resistant hypervirulent *Klebsiella pneumoniae* (CR-hvKP) can cause life-threatening infections and is therefore of global concern. Despite its importance, the evolutionary history and mechanism for the emergence of ST11 CR-hvKP are unclear. In recent years, the detection rate of ST11 CR-hvKP has increased in a teaching hospital. Based on its clonal transmission, a conjugation experiment was performed between a hvKP strain AP8555 and a ST11 CRKP strain, resulting in two ST11 CR-hvKP strains. Research had confirmed that the virulence plasmid pAP855 was horizontally transferred to the CRKP strain to form the conjugant S270-Tc, which was recombined by mobile genetic elements to evolve into the conjugant S270-Tc-R. The S270-Tc-R had high virulence, high plasmid stability, and greater adaptability. Interestingly, it had high homology to clinically prevalent ST11 CR-hvKP strains using pulsed-field gel electrophoresis (PFGE) and whole-genome sequencing (WGS). This is the first demonstration that plasmid recombination in vitro has led to the formation of ST11 CR-hvKP strains. The clinical setting is a multi-factorial and multi-selection pressure environment that may stimulate the evolution of conjugant strains in the transition period to local strains in the stable period, and surveillance is urgently needed for infection control.

**IMPORTANCE:**

The emergence of carbapenem-resistant hypervirulent Klebsiella pneumoniae (CR-hvKP) heralded the onset of a new and rapidly worsening public health disaster on a global scale. More attention has been paid to its evolutionary history and mechanism, which currently remains unclear. In this study, a conjugation experiment was performed between a hvKP strain AP8555 and a ST11 CRKP strain, resulting in two ST11 CR-hvKP strains. We had confirmed that the virulence plasmid pAP855 was horizontally transferred to the CRKP strain to form the conjugant S270-Tc, which was recombined by mobile genetic elements to evolve into the conjugant S270-Tc-R. The S270-Tc-R had high virulence, high plasmid stability, and greater adaptability. Interestingly, it had high homology to clinically prevalent ST11 CR-hvKP strains using pulsed-field gel electrophoresis and whole-genome sequencing.

## INTRODUCTION

*Klebsiella pneumoniae* (*Kp*), an opportunistic pathogen, commonly causes pneumonia, bacteremia, and urinary tract infections (1). In recent years, two major types of clinically significant pathogens, namely, hypervirulent *Kp* (hvKP) and carbapenem-resistant (CR) *Kp* (CRKP), have attracted considerable attention worldwide (2). hvKP, first identified by Liu et al. in 1986 (3), harbors a virulence plasmid that is the major pathogenic determinant of the hypermucoviscosity and hypervirulence phenotypes (4, 5). Besides, hvKP usually is sensitive to antibiotics (6). CRKP strains are generally identified with low virulence but exhibit multiple high levels of resistance to antimicrobial agents (6–8). For a long time, the multi-drug resistant (MDR) phenotypes and hypervirulence in *Kp* did not overlap (9). As a result of the rapid development of modern medical techniques for diagnosis and treatment, the extensive use of antibiotics, and more invasive tests and procedures (10), the emergence of CR-hvKP heralded the onset of a new and rapidly worsening public health disaster on a global scale, ST11 CR-hvKP accounting for the majority of cases (2, 6, 11).

*Kp* has attracted attention for its ability to acquire genetic components of resistance and virulence genes (4, 12). The plasmids containing the *ori*T finder (13)-predicted *ori*T region and genes encoding relaxase, type IV coupling proteins, and T4SS are conjugative (4, 14). It is generally believed that conjugative plasmids are mobile genetic elements responsible for the spread of resistance and virulence genes. They are commonly found in resistance plasmids and a few virulence plasmids (12). The IncF plasmid, which carries a large number of antimicrobial resistance genes (15), is a driving force in virulence plasmid mobilization (4, 16). Yang et al. reported that the mobilizable or unmobilizable virulence plasmid could be transferred with the assistance of the Incl1 plasmid (14, 17). Xu et al. (4) identified four modes of virulence plasmid mobilization: transfer with or without the conjugative IncF plasmid and fusion with the IncF plasmid via homologous recombination or two rounds of single-strand exchange at specific 28-bp fusion sites. Hybrid plasmids containing both virulence and resistance plasmids have been reported in clinical settings. Turton et al. (18, 19) identified 13 hybrid plasmids containing both virulence and resistance genes from ST147-, ST15-, ST48-, ST101-, and ST383-type strains. Gu et al. (6) discovered that ST11 CR-hvKP strains have hypervirulent, MDR, and highly transmissible characteristics. AlthoughCR-hvKP has become a hot topic (4, 6, 11), more attention has been paid to its evolutionary history and mechanism, which currently remains unclear.

## MATERIALS AND METHODS

### Bacterial strains

CRKP strains collected from the First Affiliated Hospital of Nanchang University, Jiangxi Province, China, were screened by antibiotic susceptibility testing. Primer design according to the reference: polymerase chain reaction (PCR) and sequencing technology were used to screen CR-hvKP strains. Thirty-two CR-hvKP strains were analyzed for plasmid distribution using S1 nuclease pulsed-field gel electrophoresis (S1-PFGE). *Kp* strain AP8555 (BioSample: SAMN10790857) was recovered from a clinical specimen from a patient diagnosed with hepatophyma live abscess and bloodstream infection (20). Multi-locus sequence typing identified AP8555 as ST23, and capsular typing confirmed serotype K1 via the wzi allele (20). The strain S270 (BioSample: SAMN16561282) was a clinical ST11 type CRKP strain carrying a *bla*_KPC-2_-containing plasmid. NTUH-K2044 was ST23-K1 hvKP strain containing a classical virulence plasmid pK2044 (224,152 bp) (5). For the conjugation assays (see below), the previously reported hvKP strains AP8555 and NTUH-K2044 were used as the donor strain and the ST11 CRKP strain S270 as the recipient strain. JX-CR-hvKP-4 (BioSample: SAMN16561284) was isolated by our group from a clinical specimen, a ST11-KL64 CR-hvKP strain containing five plasmids (a virulence plasmid and a *bla*_KPC_-containing plasmid).

### Antimicrobial susceptibility test

The *in vitro* drug susceptibility test was performed using a Vitek2-Compact automated microbial analyzer and the following antibiotics ampicillin, ampicillin/sulbactam, piperacillin/tazobactam, cefazolin, ceftazidime, ceftriaxone, cefepime, aztreonam, ertapenem, imipenem, meropenem, gentamicin, amikacin, ciprofloxacin, levofloxacin, co-trimoxazole, tetracycline, and tegacycline. The instrument was used and operated in accordance with the American Institute of Clinical Laboratory Standards 2020 implementation standards.

### Conjugation experiment

The conjugation experiment was performed as previously reported (4). After overnight incubation, both donor and recipient strains were grown in lysogeny broth (LB) at 220 rpm and 37°C to logarithmic growth phase (optical density at 600 nm [OD_600_], approximately 0.6). One milliliter of donor and recipient cells were washed with phosphate-buffered saline (PBS), resuspended in 20 µL of 10-mM MgSO4, mixed, and inoculated onto LB agar plates. After overnight incubation at 37°C, the bacteria were resuspended and serially diluted in PBS and plated on antibiotic-containing LB agar plates (2-µg/mL meropenem and 5-µg/mL potassium tellurite) for transconjugant selection (according to previous study [20], LB with potassium tellurite can screen virulence plasmid containing the *terW* gene, and LB with meropenem can screen antibiotic resistance plasmid carrying the *bla*_KPC-2_ gene.). The transconjugants were further validated using XbaI and S1-PFGE combined with PCR detection.

### Growth curve measurements

Overnight cultures of conjugate strain S270-Tc, S270-Tc-R and their parents were diluted to an OD_600_ of 0.01 and grown in LB broth, 2-µg/mL meropenem LB broth or 5-µg/mL potassium tellurite LB broth at 37°C for 24 h with vigorous aeration (200 rpm). The culture cell density was determined every half hour and measured using a Thermo Scientific Multiskan FC Microplate Photometer at OD_540_ (21). All experiments were repeated three times.

### *G. mellonella* larval infection assay

For the *Galleria mellonella* larval infection assay, approximately 300 mg of larvae was stored in a special box at 4°C until use. Overnight cultures were washed and diluted to 10^6^ colony-forming units/mL using PBS. Ten larvaes in each group were challenged with 10 µL of diluent, and PBS was used as a negative control. Infected larvae were incubated in sterilized Petri dishes at 37°C for 72 h, and the survival rate was recorded every 12 h (21).

### Biofilm formation

Overnight cultures were adjusted to a cell density equivalent to a 0.5 McFarland standard. Then, 200 µL of each culture (LB broth, 2-µg/mL meropenem LB broth or 5-µg/mL potassium tellurite LB broth) was transferred to a 96-well plate in triplicate. After incubation at 37°C for 24 h, the cultures were discarded. Each well was washed twice with 200-µL PBS, and the biofilms were fixed in methanol for 10 min. Subsequently, each well was stained with 1% crystal violet solution for 15 min and rinsed with PBS until colorless. Finally, biofilms were dissolved in 100 µL of 30% formic acid for 30 min, and biofilm formation was quantified by measuring the absorbance at OD540 (21).

### Quantitative determination of CPS content

The bacterial capsule polysaccharide (CPS) was quantified as previously described (22). Briefly, 500 µL of overnight cultures were mixed with 100 µL of 1% Zwittergent in 100-mM citric acid (pH 2.0) and then incubated at 50°C for 20 min. After centrifugation, 250 µL of the supernatant was added to 1 mL of cold ethanol. The mixture was incubated at 4°C for 20 min to precipitate. After centrifugation, the pellet was dried and dissolved in 200 µL of distilled water, and then 1,200 µL of 12.5-mM tetraborate in concentrated H_2_SO_4_ was added. After vigorous vortexing, the mixture was boiled for 5 min. After cooling, 20 µL of 0.15% 3-hydroxydiphenol was added. The absorbance was measured at 520 nm.

### Plasmid stability experiment

First, the conjugate strain S270-Tc-R was propagated by serial passaging for 10 days in LB broth, 2-µg/mL meropenem LB broth, or 5-µg/mL potassium tellurite LB broth. Every 12 h, 5 µL of each culture was transferred to 5 mL of fresh LB broth, 2-µg/mL meropenem LB broth, or 5-µg/mL potassium tellurite LB broth. To assess the stability of the hybrid plasmid, the fraction of plasmid-containing cells in the population was calculated daily by counting the number of colonies growing on antibiotic-free and antibiotic-containing plates (2-µg/mL meropenem and 5-µg/mL potassium tellurite). The conjugate strain S270-Tc-R was then passaged for 10 days under meropenem and potassium tellurite pressure.

### DNA sequencing and data analysis

The genomic DNA of the S270 , S270-Tc, S270-Tc-R, and JX-CR-hvKP-4 strains was extracted using the QIAamp DNA Mini Kit (Qiagen, Germany) and subsequently underwent sequencing and assembly performed by Shanghai Yuanxu Biotechnology Co. Ltd., as described in a previous study (23). A total of 5-µg genomic DNA from was sheared by g-TUBE (Covaris, USA). The sequencing library with 10-kb size was constructed using the standard PacBio RS sample preparation instructions and then sequenced on Pacific Biosciences RS II sequencing platforms (Pacific Biosciences, USA). Additionally, a 300-bp paired-end library from the same genomic DNA was prepared according to Illumina TruSeq DNA sample preparation recommendations and sequenced on HiSeq 2500 platforms (Illumina, USA) with a read length of 150 bp. The Pacbio data (10-kb fragment library, 356,001 reads) were assembled using Hierarchical Genome Assembly Process (HGAP) software (24), generating a one-contig genome and a plasmid. The assembled genome and plasmid from the Pacbio data were further proofread using HiSeq data via Bowtie2 and samtools (25, 26). Finally, a whole genome assembly without redundancy was obtained.

### Statistics

GraphPad Prism (version 9.0.1) was used to compare growth curves, relative fitness, biofilm formation ability, survival rate, and plasmid stability. Differences in survival were compared using the log-rank (Mantel–Cox) test. Absorbance values for biofilm formation between strains with and without the hybrid plasmid were compared using a *t*-test. Statistical significance was set at *P* < 0.05.

## RESULTS

### The conjugation test between hvKP strain and CRKP strain resulted in two types of conjugates

**Fig 1:**
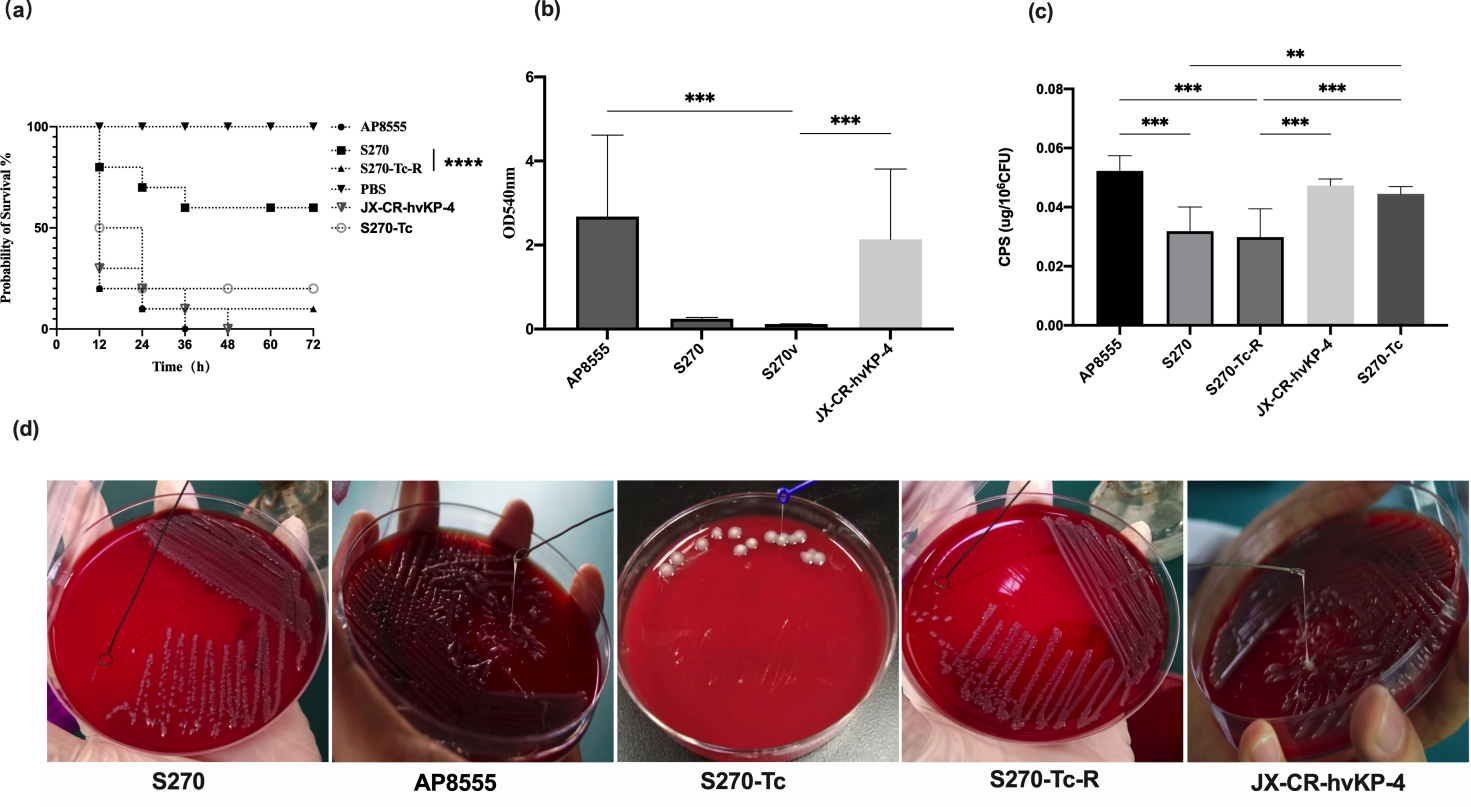
Results of virulence experiment and biofilm formation ability of AP8555 strain and S270 strain and their conjugants S270-Tc, S270-Tc-R strain, and clinical strain JX-CR-hvKP-4. (a) Virulence potential of AP8555, S270, S270-Tc, S270-Tc-R, and clinical strain JX-CR-hvKP-4 in *G. mellonella* infection model. ****P < 0.001. (b) Biofilm formation ability of strain AP8555, S270, S270-Tc, S270-Tc-R, and clinical strain JX-CR-hvKP-4. ***P < 0.001. (c) Comparison of capsular polysaccharide contents between AP8555, S270, S270-Tc, S270-Tc-R, and JX-CR-hvKP-4 strains. **P < 0.003, ***P < 0.001，ns, no difference. (d) Comparison of string test between AP8555, S270, S270-Tc, S270-Tc-R, and clinical strain JX-CR-hvKP-4. The strains AP8555, S270-Tc, and JX-CR-hvKP-4 have obvious string phenomenon, but S270-Tc-R and S270 strains are negative.

**Fig 2:**
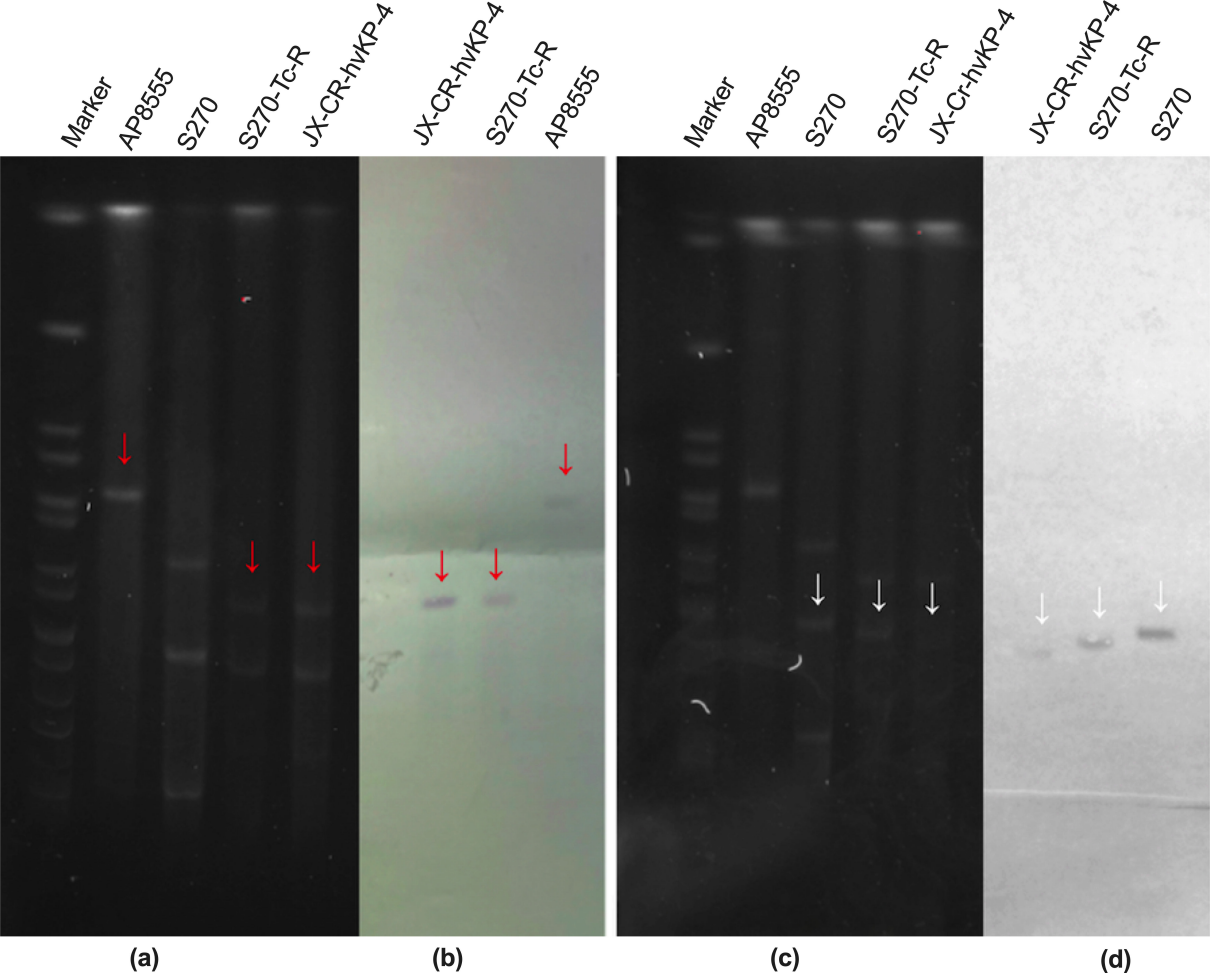
S1-PFGE and Southern blot of *Kp* conjugant strain S270-Tc-R and its parental strains (AP8555 and S270) and clinical strain (JX-CR-hvKP-4). The red arrows represent the virulence plasmid, the white arrows represent the *bla*_KPC_-_2_-containing plasmid. (**a**, c) S1-PFGE of *Kp* conjugant strain S270-Tc-R and its parental strains, clinical strains. (b) Southern blotting hybridization with a *rmpA2* specific probe. (d) Southern blotting hybridization with a *bla*_KPC_-specific probe.

In recent years, the detection rate of CRKP strains in our hospital had reached 24.93%, while CR-hvKP strains accounted for 15.97%. A total of 32 CR-hvKP isolates (Table S1) were randomly selected, of which ST11 strains accounted for 87.5% (28/32). Based on their clonal transmission, a conjugation experiment was performed. The hvKP strain AP8555 carried a novel conjugative pK2044-like virulence plasmid of approximately 357.8 kbp, and its high virulence was confirmed by *G. mellonella* larvae infection assay (Fig. 1a) (20). CRKP strain S270 was a clinical isolate of serotype K64 ST11 and carried the IncFII plasmid. The conjugation frequency between the strain AP8555 and S270 was 1.52 × 10^−4^ at 37℃, while the classical virulence plasmid pK2044 in NTUH-K2044 was not successfully conjugated. The conjugation assay produced two types of conjugates, with conjugate S270-Tc (BioSample: SAMN39449843) accounting for 20% and conjugate S270-Tc-R (BioSample: SAMN30074657) accounting for 80% on the same plate. Conjugate S270-Tc was a direct product of the virulence plasmid in horizontal transfer from the hvKP strain to the CRKP strain (Fig. S1). Another conjugate S270-Tc-R was a recombinant plasmid strain (Fig. 2; Fig. S1). Moreover, compared to clinically prevalent ST11 CR-hvKP strain, the conjugate S270-Tc-R had the same plasmid profile using S1-PFGE (Fig. S1 and S2). The whole-genome sequencing (WGS) showed they had high homology (Fig. S2).

### Phenotypic characterization of conjugate strain S270-Tc and S270-Tc-R

#### Drug resistance characteristics

Antimicrobial susceptibility testing revealed that the conjugate S270-Tc showed the same drug resistance as its parent strain S270. In addition, another conjugate S270-Tc-R was susceptible to gentamicin, amikacin, and tigecycline but resistant to other antibiotics tested (Table 1). The detection of resistance genes on the plasmid of the conjugate strains was consistent with the antimicrobial susceptibility test (Table 2).

**TABLE 1:** Minimum inhibitory concentration of S270, S270-Tc, S270-Tc-R, and JX-CR-hvKP-4 for different antibacterials

Antibacterial	AP8555		S270		S270-Tc		S270-Tc-R		JX-CR-hvKP-4	
	MIC interpretatin		MIC interpretatin		MIC interpretation		MIC interpretation		MIC interpretation	
AMP	>256	R	>32	R	>32	R	>32	R	>16	R
SAM	≦8/4	S	>32	R	>32	R	>32	R	>16/8	R
TZP	≦16/4	S	>128	R	>128	R	>128	R	>64	R
CZO	≦16	S	>32	R	>32	R	>32	R	>16	R
CAZ	≦4	S	>32	R	>32	R	>32	R	>16	R
CTRX	≦1	S	>64	R	>64	R	>64	R	>32	R
FEP	≦2	S	>32	R	>32	R	>32	R	>16	R
AZT	≦4	S	>32	R	>32	R	>32	R	>16	R
ETP	≦0.5	S	>2	R	>2	R	>2	R	>4	N/A
IPM	≦1	S	>8	R	>8	R	>8	R	>8	R
MEM	≦1	S	>16	R	>16	R	>16	R	>8	R
CN	≦4	S	>16	R	>16	R	≦4	S	≦4	S
AMK	≦16	S	>64	R	>64	R	≦16	S	>32	R
CIP	≦1	S	>4	R	>4	R	>4	R	>2	R
LEV	≦1	S	>8	R	>8	R	>8	R	>4	R
SXT	≦1	S	>4	R	>4	R	>4	R	>2/38	R
TE	≦4	S	>16	R	>16	R	>16	R		
TGC	≦1	S	≦1	S	≦1	S	≦1	S	≦2	S

**TABLE 2:** Resistance genes on plasmids of strain S270-Tc, S270-Tc-R, parental S270, and clinical strain JX-CR-hvKP-4

Plasmid	Resistance gene
pS270-1	*qnrS1,aac3_IIa-c3,aph3pp_Ib-2,aph6_Id-1,arsBCR*,*bla*_CTX_M-65_
pS270-2	*bla*_KPC-2_,*bla*_CTX-M-65_,*bla*_TEM-1_, *rmtB-1*
pS270-Tc-1	*qnrS1, aac3_IIa-c3，aph3pp_Ib-2，aph6_Id-1，arsBCR,, bla*_CTX-M-65_ *，silABCEFGPRS, pcoABCDER*
pS270-Tc-2	*bla*_CTX-M-65_*，bla*_KPC-2_*，bla*_TEM-1_, *rmtB-1*
pS270-Tc-R-2	*bla*_KPC2_,*bla*_CTX-M-14_,*bla*_SHV-12_,*bla*_TEM-1_,*aac6p-Ib-b6*
pS270-Tc-R-4	*qnrS1*
pJX4-2	*bla*_KPC-2_,*bla*_CTX-M-14_,*bla*_SHV-12_,*bla*_TEM-1_,*aac6p-Ib-b6*
pJX4-3	*qnrS1*

### Virulence characteristics

Virulence and biofilm formation were also compared between conjugates S270-Tc and S270-Tc-R, and their parents. Virulence gene analysis showed that conjugate S270-Tc-R was *rmpA*-negative, whereas conjugate S270-Tc, hvKP AP8555, was *rmpA*-positive (Table 3). The *G. mellonella* larval infection assay confirmed the conjugants S270-Tc and S270-Tc-R as a hypervirulent strain (Fig. 1a). Compared to conjugate S270-Tc, the virulence of conjugate S270-Tc-R was increased (Fig. 1a). The biofilm formation ability test showed no statistical difference between conjugate strain S270-Tc, hvKP strain AP8555, and CR-hvKP strain JX-XR-hvKP-4, all of which had strong biofilm formation ability. In addition, the conjugate strain S270-Tc-R and CRKP strain S270 were both weak in biofilm formation ability (Fig. 1b). The CPS quantification test suggested that the conjugate strain S270-Tc had a high content of CPS, as did the hvKP strain AP8555 and the CR-hvKP strain JX-XR-hvKP-4, whereas the strains S270-Tc-R and CRKP strain S270 had a low content of CPS (Fig. 1c). The conjugate S270-Tc-R and the parent strain S270 were negative in the string test, whereas the conjugate S270-Tc and the parental hvKP strain were positive (Fig. 1d).

**TABLE 3:** The virulence genes on plasmids of strain S270-Tc, S270-Tc-R, parental AP8555, S270, and clinical strain JX-CR-hvKP-4

Plasmid	Virulence gene
pAP855	*iroBCDN*, *terW*, *iucABCD*, *iutA*, *rmpA*, *rmpA2*
pS270-1	*silABCEFGPRS*
pS270-Tc-6	*iroBCDN*，*iucABCD*, *iutA*, *rmpA*, *rmpA2*, *terABCDEWXYZ*，*silABCEFGPRS*
pS270-Tc-R-1	*terABCDEXYZW*, *silABCEFGPR*, *rmpA2*, *iucABCD*, *iutA*
pJX4-1	*terABCDEXYZW*, *silABCEFGPRS*, *rmpA*, *rmpA2*, *iucABCD*, *iutA*

### Genome and plasmid analysis of conjugates S270-Tc and S270-Tc-R

The conjugation experiment between the hvKP strain and the CRKP strain resulted in two conjugates with different plasmid profiles. Phylogenetic analysis revealed that the two conjugates belonged to *Kp* and were ST11 based on multi-locus sequence typing and KL64 serotype based on capsular typing using the wzi allele. According to WGS analysis, the conjugate S270-Tc contained one chromosome and six plasmids, and the conjugate S270-Tc-R contained one chromosome and four plasmids. The conjugate S270-Tc was an intuitive product of horizontal transfer of the virulence plasmid pAP855 to the CRKP strain, whereas the S270-Tc-R strain was a product of plasmid recombination. Conjugate S270-Tc inherited all virulence and resistance genes from the parental strain, whereas the conjugate strain S270-Tc-R lost the virulence genes (*rmpA*, *iroBCDN*, and *silS*) and resistance genes (*rmtB-1*, *aac3_IIa-c3*, *aph3pp_Ib-2*, *aph6_Id-1*, and *arsBCR*) (Tables 2 and 3).

### Virulence plasmid analysis of conjugates S270-Tc, S270-Tc-R

**Fig 3:**
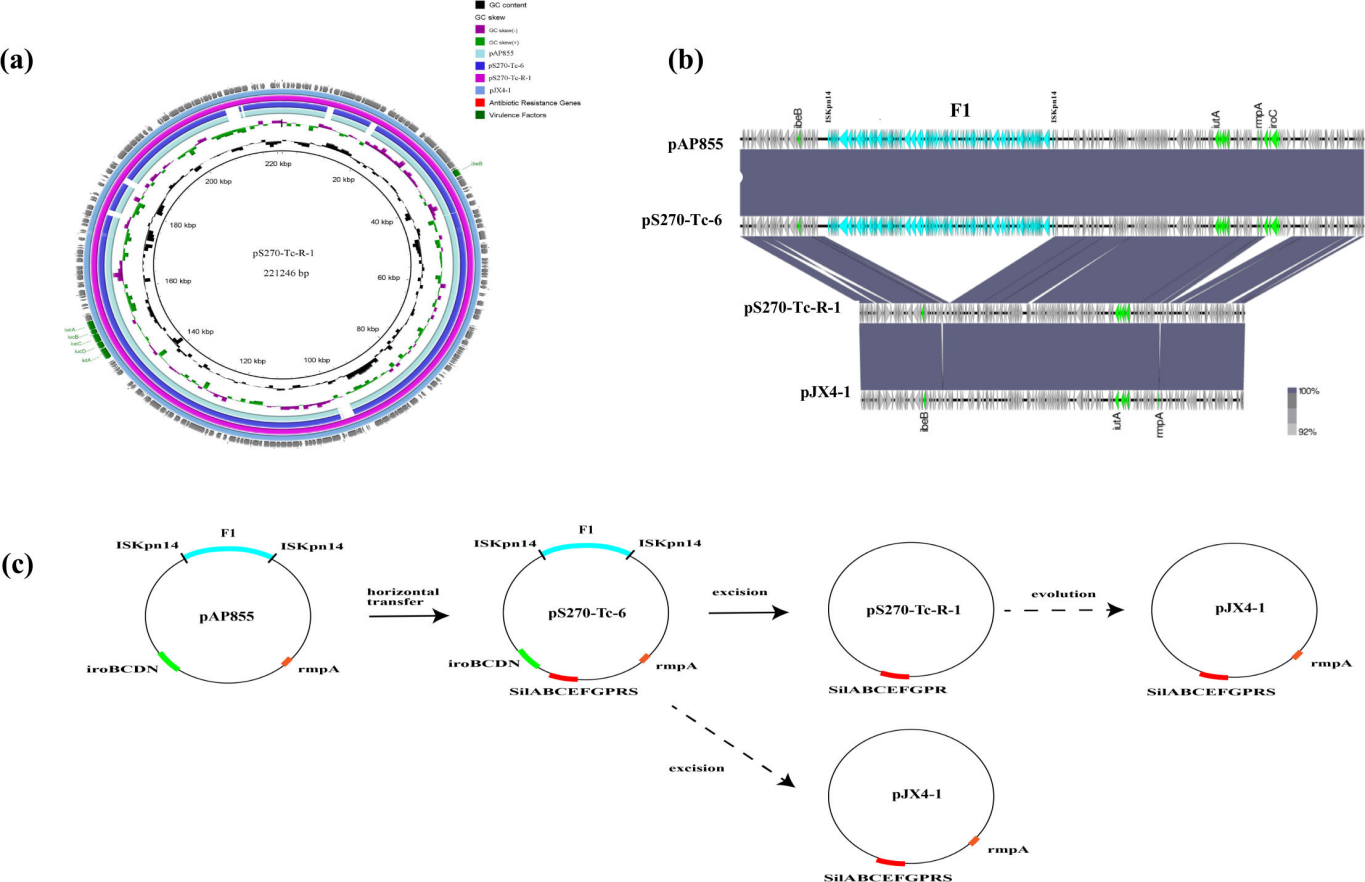
(a, b) Circular and linear comparisons of conjugant strain pS270-Tc-6, pS270-Tc-R-1 and its parental generation pAP855, and clinical strain pJX4-1. (c) Diagrams of circular transposon unit formation of conjugant strain pS270-Tc-6, pS270-Tc-R-1, and clinical strain pJX4-1.

The parental virulence plasmid pAP855 (Table 4) containing the type IV secretion system was conjugative. According to the WGS analysis, the plasmid pS270-Tc-6 (Table 4) in conjugate S270-Tc had 100% identity and coverage compared to it (Fig. 3a and b). This means that pS270-Tc-6 inherited the complete genetic information of the virulence plasmid pAP855. It also inherited the *SilABCEFGPRS* genes from the recipient strain. The plasmid pAP855 had formed the virulence plasmid pS270-Tc-R-1 (Table 4) in strain S270-Tc-R after deletion of a large fragment 1 (F1) by the insertion sequence ISKpn*14* (Fig. 3b and c). Two ISKpn*14* were identified at the excision site of plasmid pAP855. The F1 contained genes related to the IV secretion system (*TraABCDEFI*). The Southern blot located the position of the plasmid pS270-Tc-R-1 by *rmpA2* probe (Fig. 2). In addition, the plasmid pS270-Tc-R-1 did not inherit the expression of *rmpA* and *iroBCDN* genes from the donor plasmid pAP855 (Fig. 3c; Table 3).

**TABLE 4:** Strain and plasmid information from NCBI

Strain	BioSample no	Plasmid(s)	Accession no
AP8555	BioSample SAMN10790857	pAP855	accession NZ_CP035384
S270	BioSample SAMN16561282	pS270-1	accession NZ_CP064247
		pS270-2	accession NZ_CP064248
		pS270-3	accession NZ_CP064249
		pS270-4	accession NZ_CP064250
		pS270-5	accession NZ_CP064251
S270-Tc	BioSample SAMN39449843	pS270-Tc-1	accession NZ_CP142388
		pS270-Tc-2	accession NZ_CP142389
		pS270-Tc-3	accession NZ_CP142390
		pS270-Tc-4	accession NZ_CP142391
		pS270-Tc-5	accession NZ_CP142392
		pS270-Tc-6	accession NZ_CP142387
S270-Tc-R	BioSample SAMN30074657	pS270-Tc-R-1	accession NZ_CP102193
		pS270-Tc-R-2	accession NZ_CP102196
		pS270-Tc-R-3	accession NZ_CP102195
		pS270-Tc-R-4	accession NZ_CP102194
JX-CR-hvKP-4	BioSample SAMN16561284	pJX4-1	accession NZ_CP064236
		pJX4-2	accession NZ_CP064237
		pJX4-3	accession NZ_CP064238
		pJX4-4	accession NZ_CP064239
		pJX4-5	accession NZ_CP064240

### *The bla*_KPC*-2*_-*containing plasmid analysis of conjugates S270-Tc and S270-Tc-R*

**Fig 4:**
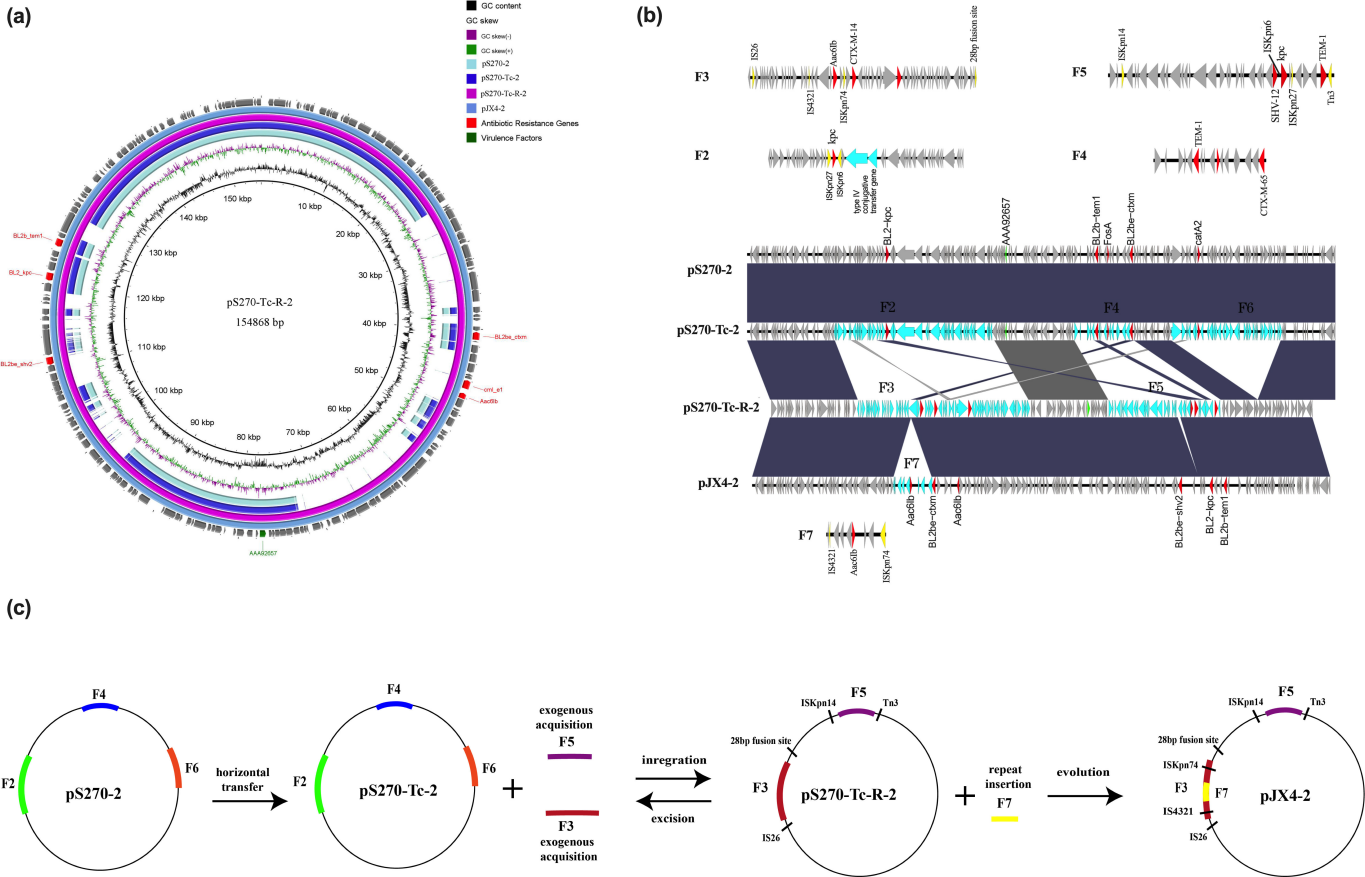
(a, b) Circular and linear comparisons of conjugant plasmid pS270-Tc-2, pS270-Tc-R-2, its parental generation pS270-2, and clinical strain pJX4- 2. (c) Diagrams of circular transposon unit formation of conjugant strains pS270-Tc-2 and pS270-Tc-R-2, and clinical strain pJX4-2.

The *bla*_KPC-2_-containing plasmid pS270-Tc-2 (Table 4) exhibited 100% identity and coverage to the plasmid pS270-2 (Table 4) in the receptor strain (Fig. 4a and b). Plasmid linear comparison analysis confirmed that they possessed consistent genetic information. The *bla*_KPC-2_-containing plasmid pS270-Tc-R-2 (Table 4) in strain S270-Tc-R was a recombinant plasmid. It was located on Southern blot using *bla*_KPC-2_ probe (Fig. 2). It exhibited 99.99% identity and 62% coverage to the pS270-2 in strain S270 (Fig. 4a and b). Fragment 3 (F3) in pS270-Tc-R-2 was integrated by IS*26* and 28-bp fusion site; it contained *bla*_CTX-M-14_ resistance gene. When compared with the corresponding position on pS270-2, it was found that genes associated with conjugation transfer (*TraBCDEFHIPSVW*) were lost. Fragment 5 (F5) in pS270-Tc-R-2 was integrated by Tn*3* and ISKpn*14*, it contained *bla*_KPC-2_ and *bla*_TEM-1_ resistance genes. (Fig. 4a, b and c).

### KP S270-Tc-R is a transitional strain developed into JX-CR-hvKP-4 strain phenotypic characterization

The antimicrobial susceptibility test suggested that the clinically prevalent strain JX-CR-hvKP-4 had wider spectrum of drug resistance information (Table 1). *G. mellonella* larval infection assay showed that strains JX-CR-hvKP-4 and S270-Tc-R had almost the same virulence. (Fig. 1a). Besides, compared with the strain S270-Tc-R, the strain JX-CR-hvKP-4 had a positive string phenotype and higher CPS content (Fig. 1c and d).

### Genome and plasmid analysis

According to the WGS analysis, the strain JX-CR-hvKP-4 had five plasmids. Its four plasmids were almost identical to those in the conjugate strain S270-Tc-R. The pJX4-1 (Table 4) was almost identical to the pS270-Tc-R-1 except expressing *rmpA* and *silS* genes (Fig. 3a, b and c; Table 2; Fig. S3). The pJX4-2 (Table 4) in clinical strain JX-CR-hvKP-4 exhibited 99.99% identity and 100% coverage to the pS270-Tc-R-2 (Fig. 4a, b and c). Fragment 7 (F7) on the pJX4-2 was the repeated insertion of the *AAC (6 ')-Ib3* gene mediated by ISKpn*74* and IS*4321* on F3. The pJX4-3 (Table 4) exhibited 100% identity and 100% coverage to the pS270-Tc-R-4 (Fig. S4a and b). Besides, the pJX4-4 (Table 4) was identical to pS270-Tc-R-3 (Table 4) (Fig. S4c and d).

### The fitness cost of conjugant strains S270-Tc and S270-Tc-R

#### Growth curve of conjugant strains S270-Tc and S270-Tc-R and their parents in different LB broths

**Fig 5:**
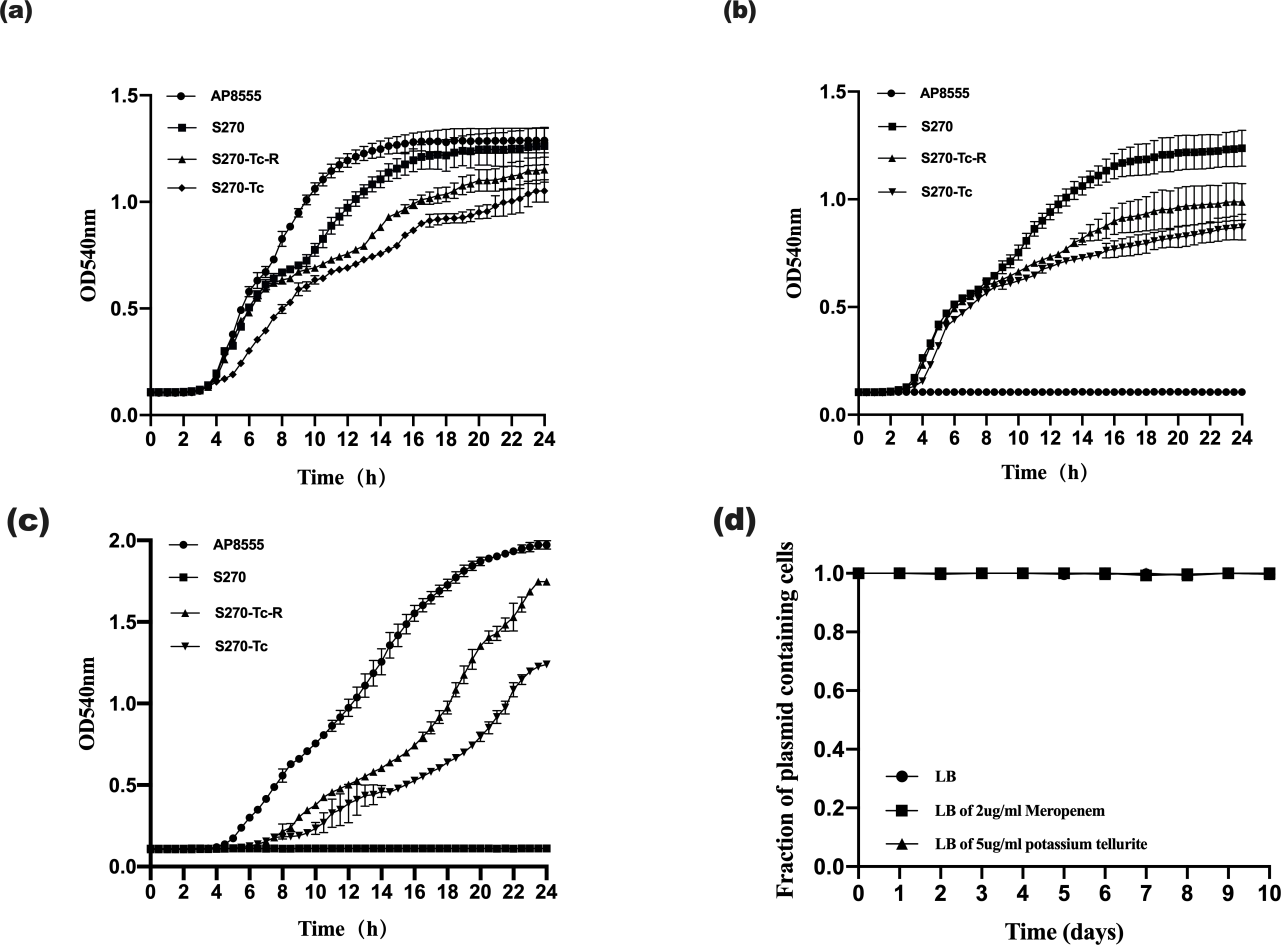
(a, b, c) Growth curves of AP8555, S270, S270-Tc, and S270-Tc-R strains in temperture of 37°C. (a) Under the condition of LB broth. (b) Under the condition of LB broth containing 5-µg/mL potassium tellurite. (c) Under the condition of LB broth containing 2-µg/mL meropenem. (d) Plasmid stability in bacterial population after serial passaging in antibiotic-free LB broth, LB broth of 2-µg/mL meropenem, and LB of 5-µg/mL potassium tellurite. Each point is the mean of three individual replicates.

The conjugant strains and their parents were subjected to fitness cost evaluation. According to the bacterial growth curves, the conjugant strains had an obvious decrease in growth rate compared to that of their parents. Under LB broth conditions, the AP8555 strain reached a growth plateau first, followed by S270 strain, S270-Tc-R strain , and, lastly, S270-Tc strain. Under conditions of LB broth containing 2-µg/mL meropenem or 5-µg/mL potassium tellurite, the conjugate strains reached the plateau more slowly than their parents. Under all conditions, S270-Tc-R strain had a better growth trend than S270-Tc strain (Fig. 5a, b and c). These data implied that the conjugants had a weaker growth trend than their parents in an ideal or a single selection environment, but the clinical setting is a multi-factorial and multi-selection pressure environment that contributes to the widespread dissemination of S270-Tc-R strain.

### The stability of conjugate strain S270-Tc-R

To evaluate the stability of conjugate strain S270-Tc-R, it was propagated in antibiotic-free LB broth, LB broth containing 2-µg/mL meropenem, or 5-µg/mL potassium tellurite for 10 days. The hybrid plasmid was stably maintained in at least 98% of cells in each medium (Fig. 5d). The high persistence of the hybrid plasmid in different LB broths was largely a consequence of plasmid recombination.

## DISCUSSION

**Fig 6:**
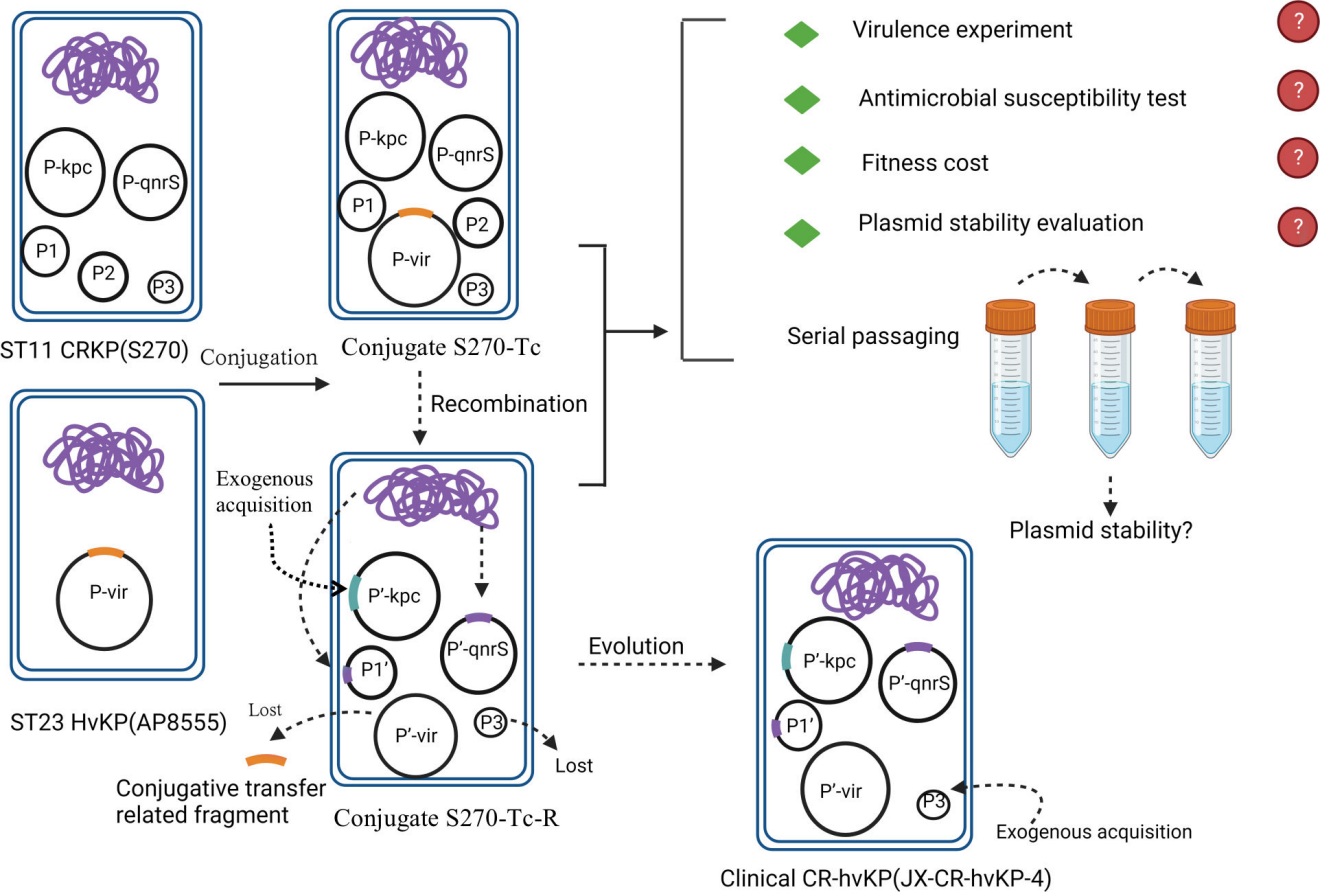
Schematic diagram of research process. The conjugant strain was produced by the conjugation experiment of hvKP and ST11 CRKP. The purple curves represent chromosome. The orange color represents conjugative transfer-related genes fragments. The blue color represents exogenous acquisition genes fragments. The conjugant strain was subjected to fitness evaluation and serial passaging under LB broth, 2-µg/mL meropenem LB broth, and 5-µg/mL potassium tellurite LB broth for 10 days.

The emergence of CR-hvKP in China has posed a great threat to public health, especially in the highly transmissible ST11 clone (2, 6, 11). In recent years, the detection rate of ST11 CR-hvKP has been increasing in our hospital. To clarify the formation mechanism of CR-hvKP, a conjugation experiment was carried out between an hvKP strain and a ST11 CRKP strain. The strain AP8555 carried a novel conjugative pK2044-like virulence plasmid which could be conjugated to *K. quasipneumoniae* ATCC700603 and *Escherichia coli* J53 (20). The conjugation test produced two different plasmid profiles ST11 CR-hvKP (Fig. 6). The conjugate strain S270-Tc was a product of horizontal transfer of virulence plasmid pAP855, and its six plasmids had 100% identity and coverage with the plasmids in the parental strains (Fig. 6). Interestingly, the conjugate strain S270-Tc-R was a product of plasmid recombination, and it had high homology with the clinically prevalent ST11 CR-hvKP strains (Fig. 6).

Mobile genetic elements play an important role in plasmid recombination and evolution (27–29). In this study, the pAP855-deleted transfer-related fragments contained type IV secretion system by the insertion sequence ISKpn*14* and then evolved into pS270-Tc-R-1 . The process involved the loss of iron uptake system-related gene *iroBCDN* and the CPS synthesis regulatory gene *rmpA*. It also involved the acquisition of the silver resistance gene *silABCEFGPR* on the recipient bacterium. The loss of *rmpA* had led to a negative string test and low CPS quantification of the conjugate S270-Tc-R. This is consistent with previous reports that the *rmpA* and *rmpA2* genes regulate the mucoid phenotype, promoting the synthesis of CPS (30, 31). Deletion of *rmpA* reduced K2 CPS biosynthesis, resulting in decreased colony mucoidy and virulence in mice (32). The virulence plasmid pJX4-1 of the clinical strain JX-CR-hvKP-4 had the complete gene mentioned above, which may had been formed by the deletion of the F1 and *iroNBCDN* by pS270-Tc-6, or by the acquisition of *rmpA* and *silS* by pS270-Tc-1 during the evolutionary process.

Plasmid recombination events have been reported in many studies. A fusion event involving homologous recombination between a 241-bp homologous region located in the IncFIA plasmid and the hypervirulence-encoding plasmid has been reported (33). In this study, the *bla*_KPC-2_-containing plasmid pS270-Tc-R-2 was a recombinant plasmid. Its formation involved IS*26*, ISKpn*14* insertion sequences, Tn*3* transposon, and 28-bp fusion site. IS*26* and Tn*3* family transposases had been reported to be involved in the recombination of drug-resistant plasmids (27, 28, 34, 35), which may be the basis for the formation of pS270-Tc-R-2. In addition, the formation of pS270-Tc-R-2 involved the acquisition of exogenous sequences, which may be related to maintenance of the plasmid stability. The *bla*_SHV-12_ gene on pS270-Tc-R-2 may be derived from *bla*_SHV-11_ on the chromosome of the recipient bacterium S270. Chromosomal integration has been shown to be associated with MDR. Zhan et al. (36)reported that plasmid and chromosome integration events accompany the formation of *bla*_IMP_ transposon, facilitating the spread of drug resistance genes. Wyres, K.L et al. (37) demonstrated that frequent chromosomal recombination generates extensive diversity in surface polysaccharide locus, resulting in a high diversity of MDR clonal strains. Hudson, C.M et al. (38) reported that the gene *bla*_CTX-M-15_ in the plasmid backbone was integrated into the chromosome via ISEcp*1*. Analysis of the *bla*_KPC-2_-containing plasmid pS270-Tc-R-2 revealed that the *bla*_SHV-12_ gene may be derived from *bla*_SHV-11_ on the chromosome of the recipient bacterium S270.

Plasmids impose a fitness cost on their hosts (39), the conjugate strains S270-Tc and S270-Tc-R showed a weaker growth trend than their parents in an ideal or single selection environment and had a fitness cost in pairwise competition experiment. This is consistent with studies reporting fitness effects after acquisition of the MDR plasmid on host bacteria (21, 39). In contrast, the plasmid paradox showed that plasmids cannot be retained without selective pressure (40). The clinical setting is a multi-factorial and multi-selection pressure environment that contributes to maintaince of plasmid stability and widespread dissemination of the experimentally engineered ST11 CR-hvKP strain.

Plasmid persistence in the natural host is largely a consequence of the long-term co-evolution of host and plasmid (21, 41). A study reported that plasmid loss is determined by transcription–replication conflicts modulated by environmental conditions (42). Alonso-Del Valle et al. (43) have shown that variability in plasmid fitness effects contributes to plasmid persistence in bacterial communities. In this study, the high plasmid stability of the conjugate strain S270-Tc-R may be explained by the fact that plasmid recombination was more stable than simple horizontal transfer.

Overall, this is the first demonstration that plasmid recombination *in vitro* has led to the formation of ST11 CR-hvKP strains. Our research had shown that the capture of mobile genetic elements promotes plasmid recombination and evolution. The CR-hvKP strain formed in the laboratory had high virulence, high plasmid stability, and greater adaptability.

### Conclusion

Capture of mobile genetic elements following intercellular conjugation of MDR and virulence plasmids promotes the production of ST11-KL64 CR-hvKP. Given its high virulence, high plasmid stability and increased adaptability, surveillance is urgently needed for infection control.

## Data Availability

The complete genome sequences of Kp AP8555, S270, and JX-CR-hvKP-4 were deposited in the National Center for Biotechnology Information (NCBI) BioSample repository under the BioSample numbers SAMN10790857, SAMN16561282 , and SAMN16561284. The genome sequence of the conjugate strains S270-Tc and S270-Tc-R were deposited in the NCBI BioSample repository under the BioSample numbers SAMN39449843 and SAMN30074657. The accession numbers of all the other sequences analyzed during the current study are included in this manuscript and available in the NCBI Nucleotide database.

## SUPPLEMENTAL MATERIAL

Fig. S1 spectrum.01347-24-s0001.tif S1-PFGE of Klebsiella pneumoniae conjugant S270-Tc, S270-Tc-R and its parental strains (AP8555, S270), clinical strain JX-CR-hvKP-4.

Fig. S2 spectrum.01347-24-s0002.png (a)(b) S1-PFGE and PFGE of Klebsiella pneumoniae hybrid strain S270-Tc-R and its parental strains (AP8555, S270), clinical strains (JX-CR-hvKP-4, KP1-10). (c) Gene circular comparision of hybrid strain S270-Tc，S270-Tc-R and its parental gengeration, clinical strain JX-CR-hvKP-4.

Fig. S3 spectrum.01347-24-s0003.tif Linear comparision of conjugant plasmid pS270-Tc-R-1 and clinical strain plasmid pJX4-1.

Fig. S4 spectrum.01347-24-s0004.tiff (a)(b) Linear comparision of conjugant plasmid pS270-Tc-R-3, pS270-Tc-4, pS270-4 and clinical strain plasmid pJX4-4. (c)(d) Linear comparision of conjugant plasmid pS270-Tc-R-4, pS270-Tc-1, pS270-1 and clinical strain plasmid pJX4-3.

Table S1 spectrum.01347-24-s0005.docx Informations of CR-hvKP strains.

ASM does not own the copyrights to Supplemental Material that may be linked to, or accessed through, an article. The authors have granted ASM a non-exclusive, world-wide license to publish the Supplemental Material files. Please contact the corresponding author directly for reuse.
