# Supplementary figures and images for: Capture of mobile genetic elements following intercellular conjugation promotes the production of ST11-KL64 CR-hvKP
Source: Microbiol Spectr. 2025 Feb 3;13(3):e01347-24. doi: 10.1128/spectrum.01347-24 (PMC11878025; doi:10.1128/spectrum.01347-24)

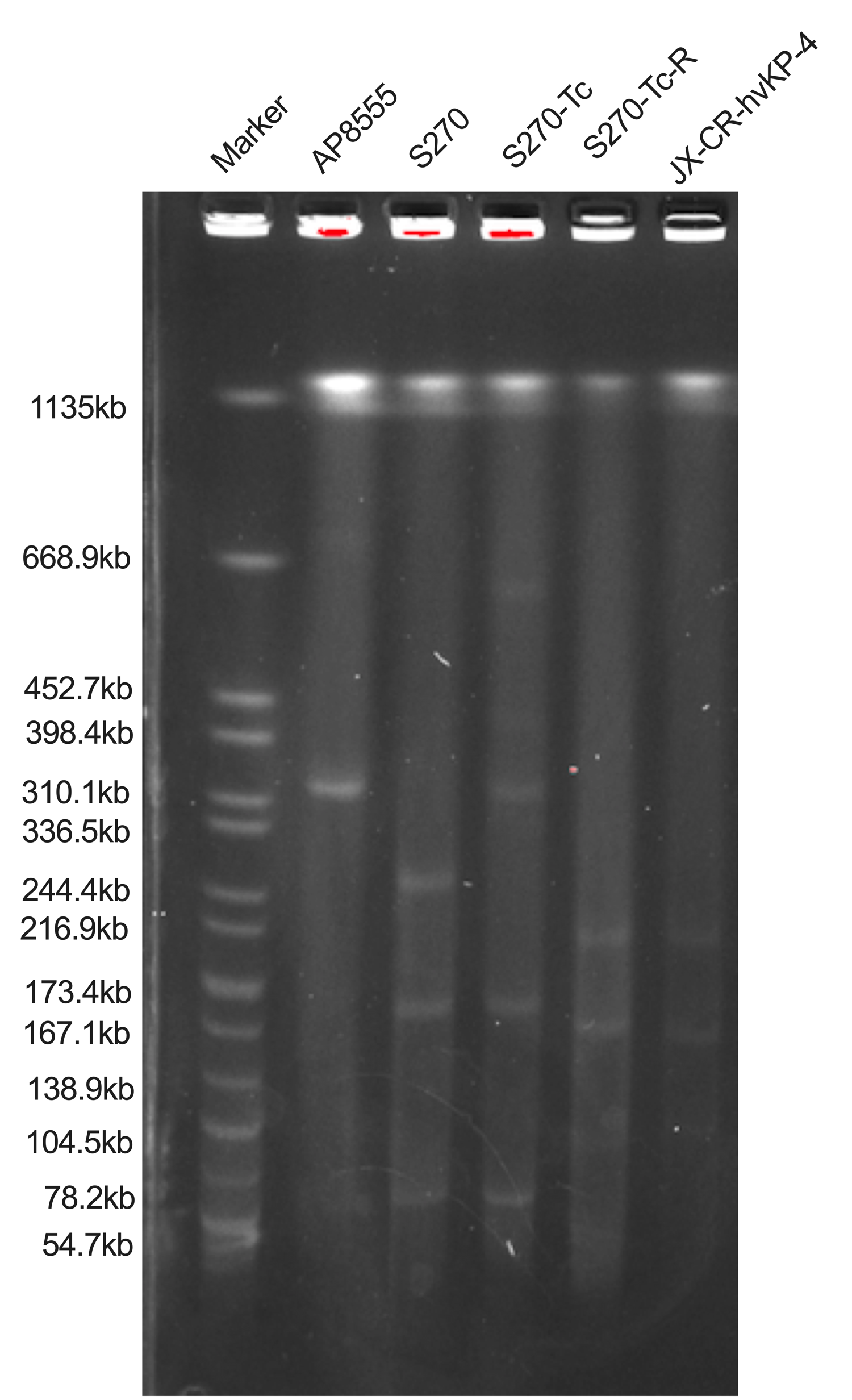

Supplement: Fig. S1 — S1-PFGE of Klebsiella pneumoniae conjugant S270-Tc, S270-Tc-R and its parental strains (AP8555, S270), clinical strain JX-CR-hvKP-4. [file spectrum.01347-24-s0001.tif]

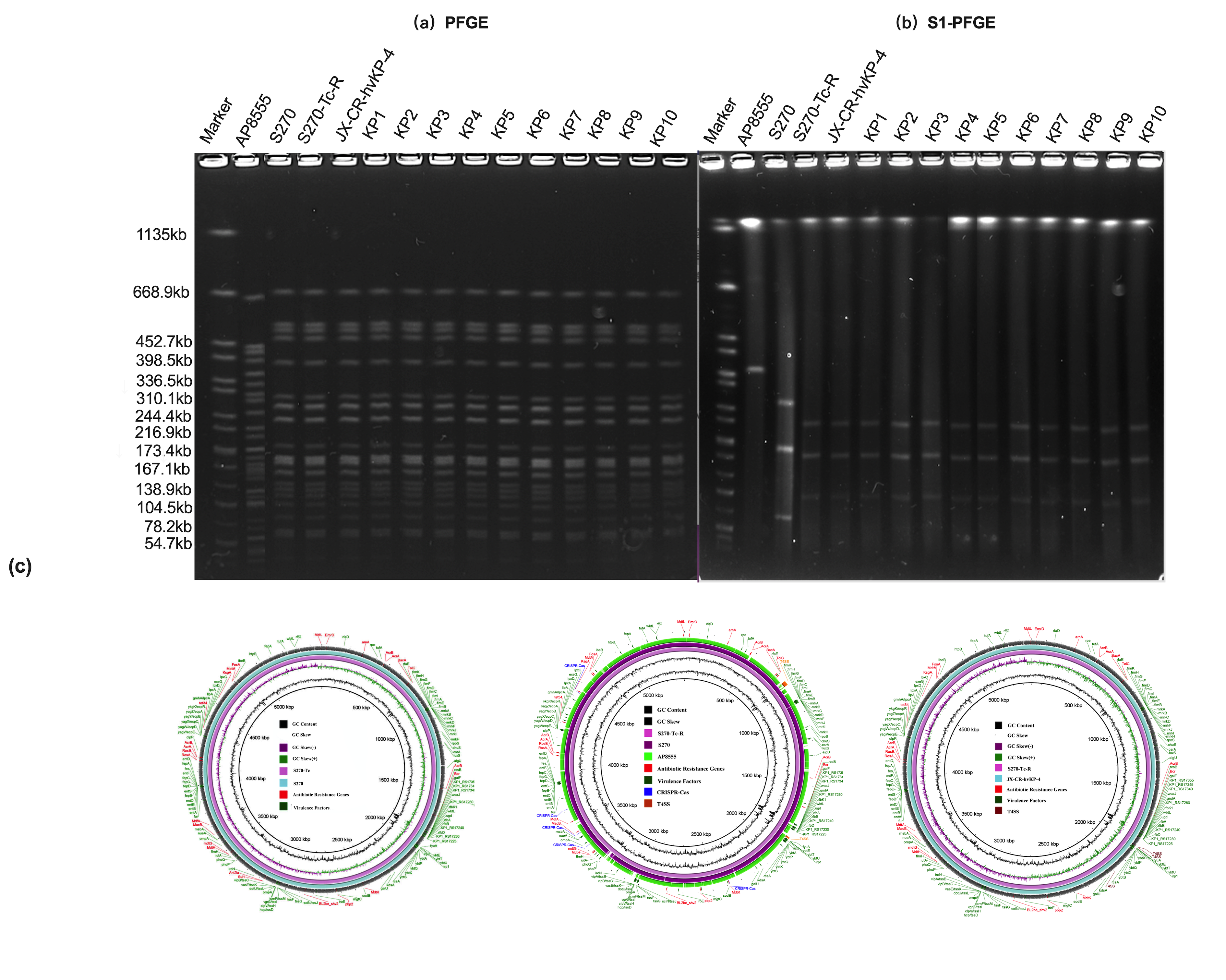

Supplement: Fig. S2 — (a)(b) S1-PFGE and PFGE of Klebsiella pneumoniae hybrid strain S270-Tc-R and its parental strains (AP8555, S270), clinical strains (JX-CR-hvKP-4, KP1-10). (c) Gene circular comparision of hybrid strain S270-Tc，S270-Tc-R and its parental gengeration, clinical strain JX-CR-hvKP-4. [file spectrum.01347-24-s0002.png]

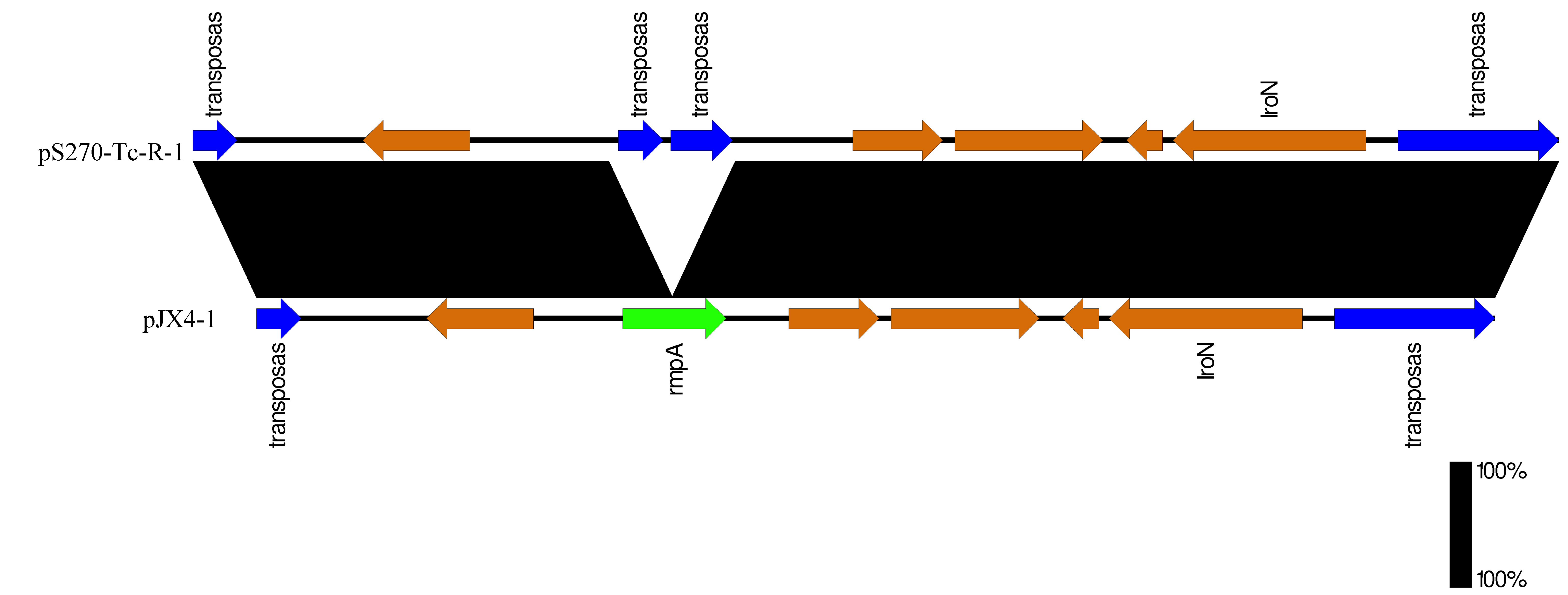

Supplement: Fig. S3 — Linear comparision of conjugant plasmid pS270-Tc-R-1 and clinical strain plasmid pJX4-1. [file spectrum.01347-24-s0003.tif]

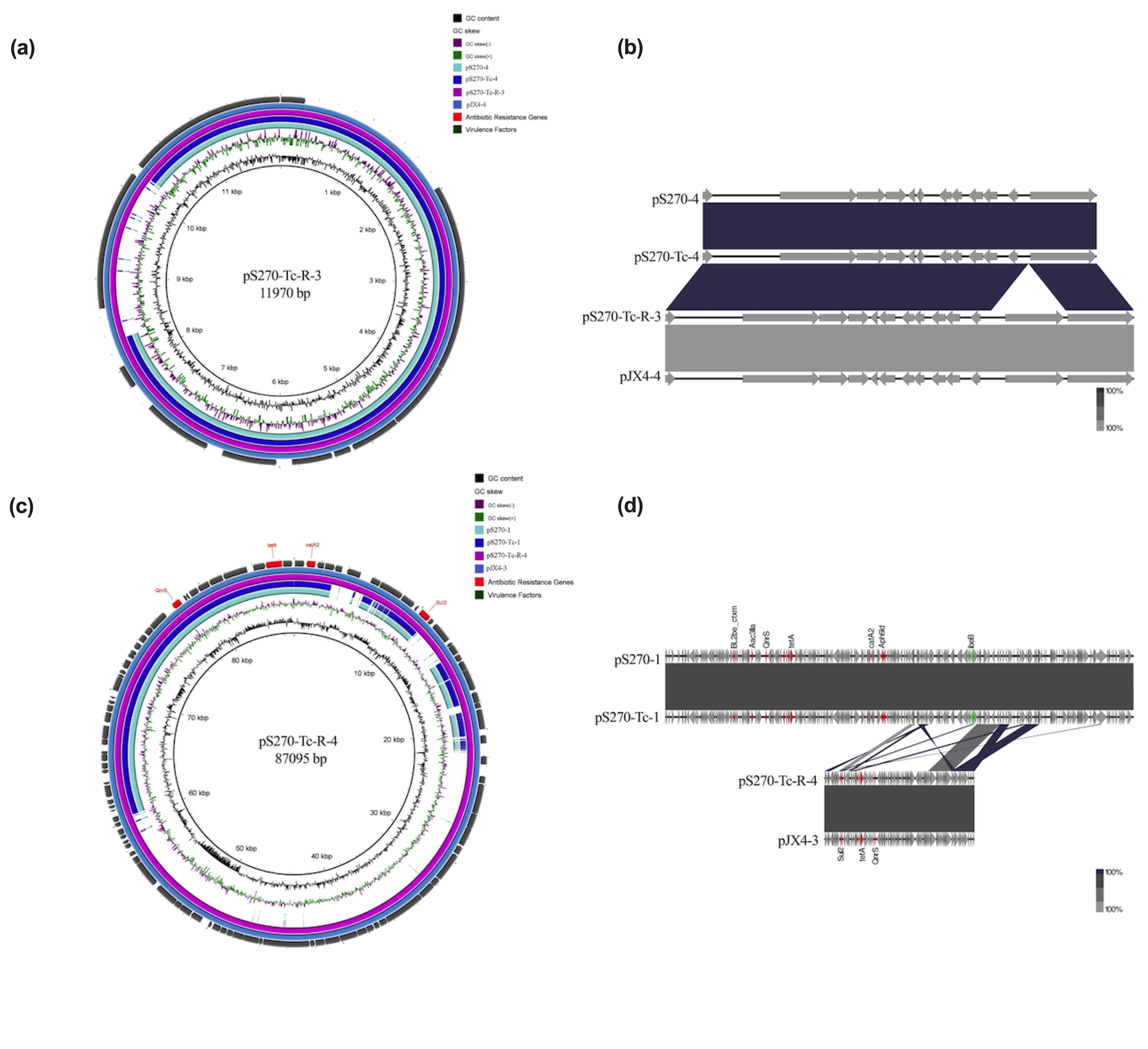

Supplement: Fig. S4 — (a)(b) Linear comparision of conjugant plasmid pS270-Tc-R-3, pS270-Tc-4, pS270-4 and clinical strain plasmid pJX4-4. (c)(d) Linear comparision of conjugant plasmid pS270-Tc-R-4, pS270-Tc-1, pS270-1 and clinical strain plasmid pJX4-3. [file spectrum.01347-24-s0004.tiff]
