# Supplementary material for: Capture of mobile genetic elements following intercellular conjugation promotes the production of ST11-KL64 CR-hvKP
Source: Microbiol Spectr. 2025 Feb 3;13(3):e01347-24. doi: 10.1128/spectrum.01347-24 (PMC11878025; doi:10.1128/spectrum.01347-24)
Supplement: Table S1 — Informations of CR-hvKP strains. [file spectrum.01347-24-s0005.docx]

**Supplementary table1：Informations of CR-hvKP strains**

| Strain ID | Sequnce type | Source | Capsule | String  phenomenon | Virulence genes | Resistance genes |
| --- | --- | --- | --- | --- | --- | --- |
| KP1 | 11 | sputum | KL64 | positive | *iucA, iutA,*  *mrkD,rmpA, rmpA2* | *aac6p, aph3p,*  *bla*_CTX_, *bla*_KPC_,  *bla*_SHV_, *bla*_TEM,_  *qnrS* |
| KP2 | 11 | blood | KL64 | positive | *iucA, iutA,*  *mrkD, rmpA, rmpA2* | *aac6p*, *bla*_CTX,_  *bla*_KPC,_ *bla*_SHV,_  *bla*_TEM_, *qnrS* |
| KP3 | 11 | blood | KL64 | positive | *iucA, iutA,*  *mrkD,rmpA，*  *rmpA2* | *aac6p*, *bla*_CTX,_  *bla*_KPC_, *bla*_SHV_,  *bla*_TEM_, *qnrS* |
| KP4 | 11 | blood | KL64 | positive | *iucA, iutA,*  *mrkD, rmpA,*  *rmpA2* | *bla*_KPC_, *bla*_SHV_,  *qnrS* |
| KP5 | 11 | ascites | KL64 | positive | *iucA, iutA,*  *mrkD, rmpA, rmpA2* | *ant3pp*, *bla*_CTX_, *bla*_KPC_, *bla*_TEM_,  *qnrS* |
| KP6 | 11 | sputum | KL64 | positive | *iucA, iutA,*  *mrkD, rmpA, rmpA2* | *bla*_KPC_, *bla*_SHV_,  *qnrS* |
| KP7 | 11 | sputum | KL64 | negative | *iucA, iutA,*  *mrkD, rmpA2* | *bla*_CTX_, *bla*_KPC_,  *bla*_SHV_, *bla*_TEM_,  *qnrS* |
| KP8 | 11 | urine | KL64 | positive | *iucA, iutA,*  *mrkD, rmpA, rmpA2* | *aac6p*, *bla*_CTX_,  *bla*_KPC_, *bla*_SHV_,  *qnrS* |
| KP9 | 11 | sputum | KL64 | positive | *iucA, iutA,*  *mrkD, rmpA,*  *rmpA2* | *bla*_CTX_,*bla*_KPC_,  *bla*_SHV_, *qnrS* |
| KP10 | 11 | sputum | KL64 | positive | *iucA, iutA,*  *mrkD, rmpA,*  *rmpA2* | *aac6p*, *bla*_CTX_,  *bla*_KPC_, *bla*_SHV_,  *bla*_TEM_, *qnrS* |
| KP11 | 11 | blood | KL64 | positive | *iucA, iutA,*  *mrkD, rmpA,*  *rmpA2* | *bla*_CTX_, *bla*_KPC_,  *bla*_SHV_, *qnrS* |
| KP12 | 11 | sputum | KL64 | negative | *iucA, iutA,*  *mrkD, rmpA2* | *bla*_CTX_, *bla*_KPC_,  *bla*_SHV_, *bla*_TEM_,  *qnrS* |
| KP13 | 11 | blood | KL64 | positive | *iucA, iutA,*  *mrkD, rmpA, rmpA2* | *ant3p*, *bla*_CTX_,  *bla*_KPC_, *bla*_TEM_,  *qnrS* |
| KP14 | 11 | catheter site | KL64 | positive | *iucA, iutA,*  *mrkD, rmpA，*  *rmpA2* | an*t3pp*, *bla*_CTX_, *bla*_KPC_, *bla*_SHV_,  *bla*_TEM_, *qnrS* |
| KP15 | 11 | catheter tip | KL64 | positive | *iucA, iutA,*  *mrkD, rmpA, rmpA2* | *ant3pp*,*bla*_CTX_,  *bla*_KPC_, *bla*_TEM_,  *qnrS* |
| KP16 | 23 | sputum fluid | K1 | positive | *iucA, iutA,*  *mrkD, iroN,*  *rmpA, rmpA2* | *bla*_KPC_, *bla*_SHV_ |
| KP17 | 11 | blood | K64 | positive | *iucA, iutA,*  *mrkD, rmpA, rmpA2* | *ant3pp,* *bla*_CTX_, *bla*_KPC_, *bla*_SHV_,  *bla*_TEM_ |
| KP18 | 11 | secretion | KL64 | negative | *iucA, iutA,*  *mrkD, rmpA2* | *bla*_CTX_, *bla*_KPC_,  *bla*_SHV_, *bla*_TEM_ |
| KP19 | 11 | blood | KL64 | negative | *iucA, iutA,*  *mrkD, rmpA2* | *bla*_CTX_, *bla*_KPC_,  *bla*_SHV_, *bla*_TEM_ |
| KP20 | 11 | sputum | KL64 | positive | *iucA, iutA,*  *mrkD, rmpA, rmpA2* | *aac6p*, *bla*_CTX_,  *bla*_KPC_, *bla*_SHV_,  *bla*_TEM_, *qnrS* |
| KP21 | 23 | blood | K1 | positive | *iucA, iutA,*  *mrkD, rmpA, rmpA2* | *bla*_CTX_, *bla*_KPC_,  *bla*_SHV_, *qnrS* |
| KP22 | 11 | sputum | KL64 | negative | *iucA, iutA,*  *mrkD, rmpA2* | *bla*_CTX_, *bla*_KPC_,  *bla*_SHV_, *bla*_TEM_,  *qnrS* |
| KP23 | 11 | blood | KL64 | positive | *iucA, iutA,*  *mrkD, rmpA, rmpA2* | *ant3p*, *bla*_CTX_,  *bla*_KPC_, *bla*_TEM_,  *qnrS* |
| KP24 | 11 | catheter site | KL64 | positive | *iucA, iutA,*  *mrkD, rmpA，*  *rmpA2* | *ant3pp*, *bla*_CTX_, *bla*_KPC_, *bla*_SHV_, *bla*_TEM_, *qnrS* |
| KP25 | 11 | catheter tip | KL64 | positive | *iucA, iutA,*  *mrkD, rmpA, rmpA2* | *ant3pp*, *qnrS bla*_CTX_, *bla*_KPC_, *bla*_TEM_ |
| KP26 | 23 | blood | K1 | positive | *iucA, iutA,*  *mrkD, iroN,*  *rmpA,rmpA2* | *bla*_KPC_, *bla*_SHV_ |
| KP27 | 11 | blood | K64 | positive | *iucA, iutA,*  *mrkD, rmpA, rmpA2* | *ant3pp*, *bla*_CTX,_ *bla*_KPC_, *bla*_SHV_,  *bla*_TEM_ |
| KP28 | 11 | secretion | KL64 | negative | *iucA, iutA,*  *mrkD, rmpA2* | *bla*_CTX_, *bla*_KPC_, *bla*_SHV_*, bla*_TEM_ |
| KP29 | 11 | blood | KL64 | negative | *iucA, iutA,*  *mrkD, rmpA2* | *bla*_CTX_, *bla*_KPC_, *bla*_SHV_, *bla*_TEM_ |
| KP30 | 11 | sputum | KL64 | positive | *iucA, iutA,*  *mrkD, rmpA, rmpA2* | *aac6p*, *bla*_CTX_,  *bla*_KPC_, *bla*_SHV_, *bla*_TEM_, *qnrS* |
| KP31 | 23 | blood | K1 | positive | *iucA, iutA,*  *mrkD, rmpA, rmpA2* | *bla*_CTX_, *bla*_KPC_, *bla*_SHV_, *qnrS* |
| KP32 | 11 | sputum | KL64 | negative | *iucA, iutA,*  *mrkD, rmpA2* | *bla*_CTX_, *bla*_KPC_, *bla*_SHV_, *bla*_TEM_,  *qnrS* |
